# Modus Operandi of Chloroform

**Published:** 1859-06

**Authors:** 


### MODUS OPERANDI OF CHLOROFORM.

Dr. F. Piossek read before the Physiological Society of Greisswald, an account of experiments with chloroform, made under the direction of Prof. Hunefield, which seems to establish the following conclusions as to the *modus operandi* of chloroform, beyond a doubt:

*Chloroform produces anæsthesia by abstracting from the blood* some of the *oxygen* necessary to the continuance of the organic process, thus causing *impaired nutrition* of the central organs and nerves; hence the insensibility of the sensatory, and the relaxation of the motory nerves.

The oxygen of the blood probably combines with the carbon (liberated by the decomposition of the chloroform,) to form carbonic acid, while the chlorine and water of the chloroform probably form hydrochloric acid, etc. Into what combinations this hydrochloric acid may then enter with the ingredients of the blood, is yet unknown.

The other anæsthetics, ether, amylene, etc., act similarly, and their *modus operandi* may be compared to the narcotizing or asphyxiating action of carbonic acid on nitrous oxide.

